# Laparoscopic Resection of a Gastric Lipoma Presenting With Gastrointestinal Bleeding: A Case Report

**DOI:** 10.7759/cureus.101554

**Published:** 2026-01-14

**Authors:** José Pedro Fernandes, José Paulo Couto, João Mendes, Telma Rodrigues Brito, Eduardo Vasconcelos

**Affiliations:** 1 General Surgery, Unidade Local de Saúde do Alto Minho, Viana do Castelo, PRT

**Keywords:** bleeding lipoma, gastric lipoma, giant lipoma, laparoscopic gastrectomy, upper gastrointestinal bleeding

## Abstract

Gastric lipomas are uncommon benign tumours that rarely cause significant gastrointestinal bleeding. We present the case of a 66-year-old woman who experienced severe anaemia secondary to an ulcerated gastric lipoma. Despite initial endoscopic attempts at haemostasis, surgical intervention was required. Histopathology confirmed a large, ulcerated submucosal lipoma measuring 85 mm.

This case underscores the diagnostic challenges posed by large gastric submucosal tumours and highlights the importance of considering lipomas in the differential diagnoses of gastrointestinal bleeding. Surgical resection remains the definitive treatment for symptomatic or complicated gastric lipomas.

## Introduction

Gastric lipomas are rare, benign mesenchymal tumours originating from adipose tissue within the gastric submucosa or muscularis propria, representing less than 1% of gastric tumours and 5% of gastrointestinal lipomas [1,2]. Most are small and asymptomatic, often incidentally discovered during imaging or endoscopy. However, larger lesions, especially those exceeding 2 cm, may present with nonspecific symptoms such as abdominal discomfort, nausea, or less frequently, gastrointestinal bleeding due to ulceration [3], intussusception or gastric outlet obstruction. Some diagnostic hallmarks are CT fat attenuation and endoscopic features like the tenting sign and cushion sign [3]. Differentiating gastric lipomas from other submucosal tumours, notably gastrointestinal stromal tumours (GISTs), is crucial for appropriate management [4]. Depending on size, location and symptoms, gastric lipomas may require no intervention or can be endoscopically or surgically resected [4].

This report describes a case of a giant gastric lipoma presenting with active bleeding and discusses diagnostic and therapeutic considerations.

## Case presentation

A 66-year-old woman presented to the Emergency Department with a five-day history of weakness, nausea and diarrhoea. The patient had no comorbidities and no history of nonsteroidal anti-inflammatory drugs (NSAIDs), anticoagulants, or antiplatelets use. She exhibited hypotension (BP 97/53 mmHg), tachycardia (HR 106 bpm), skin pallor, no abdominal pain or palpable masses and a digital rectal exam consistent with melena. Her laboratory results showed a haemoglobin level of 5 g/dL and a haematocrit of 14.8%, with no other significant abnormalities. She was stabilized with intravenous fluids and received a transfusion of three units of packed red blood cells.

**Figure 1:**
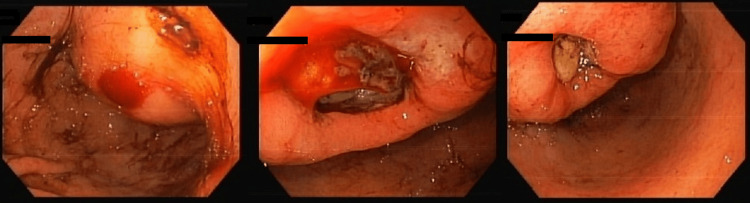
Upper gastrointestinal endoscopy showing ulcerated gastric lesion Large submucosal mass of the gastric antrum with two ulcerated areas, one of which has oozing bleeding (Forrest Ib), suspicious for gastrointestinal stromal tumour (GIST).

An urgent upper gastrointestinal endoscopy revealed a gastric cavity filled with blood and clots, along with a lesion in the gastric antrum, suggestive of an ulcerated GIST, with two ulcerated areas (Figure 1), one of which exhibited active low-flow bleeding (Forrest Grade Ib). Endoscopic haemostasis was attempted with epinephrine injection and argon plasma coagulation, but without success.

**Figure 2:**
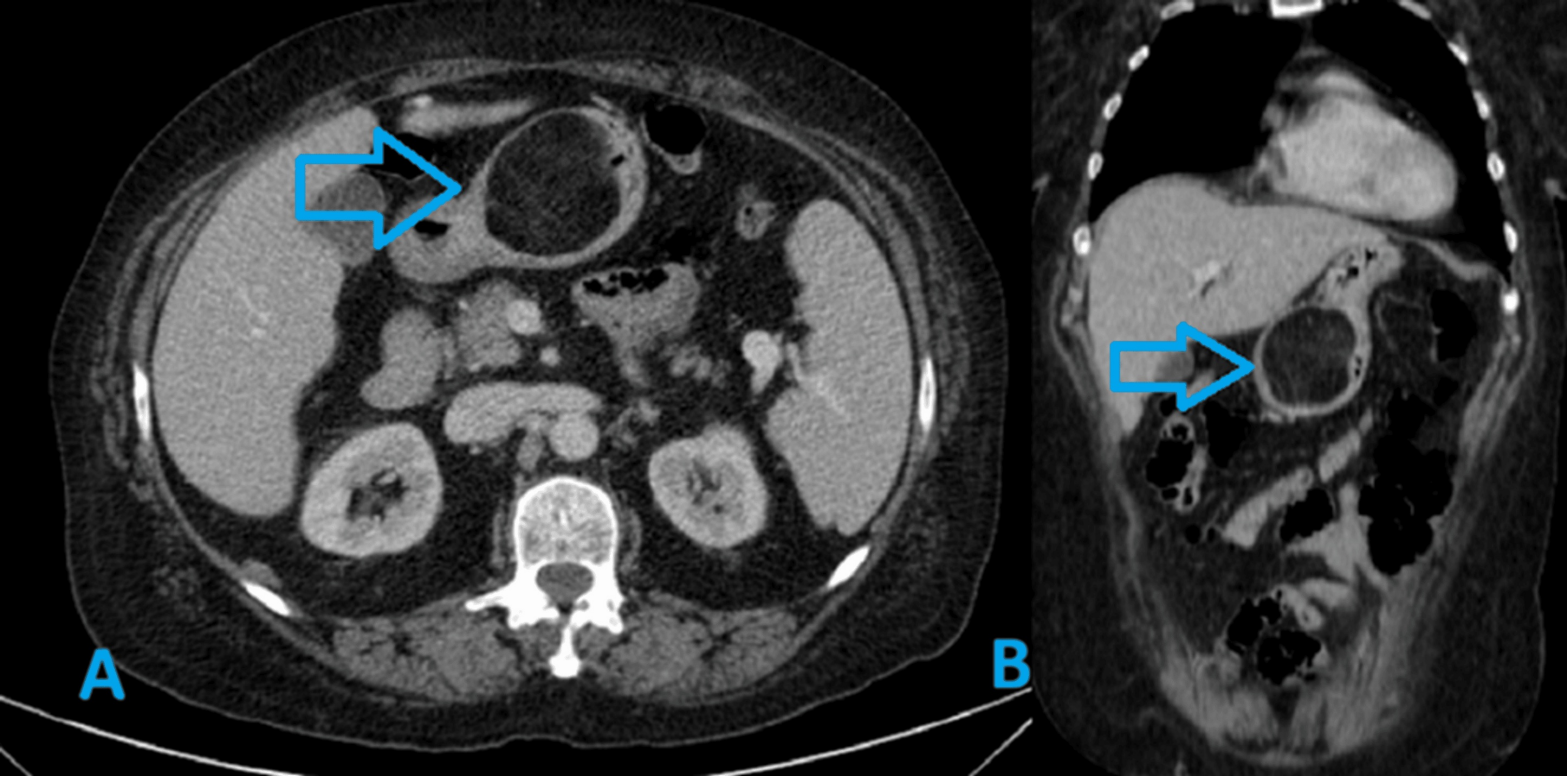
CT scan showing a lipomatous gastric lesion Axial (A) and coronal (B) views demonstrating a lipomatous gastric lesion (blue arrow) measuring approximately 56 mm originating from the greater curvature of the antrum, with attenuation value of -80 Hounsfield units (HU).

A CT scan showed a lipomatous mass on the greater curvature of the antrum measuring 56 mm, suggesting a lipomatous lesion or GIST, without secondary lesions (Figure 2).

Since effective haemostasis could not be achieved during upper GI endoscopy, surgical intervention was performed the following day. Due to the initial suspicion of an ulcerated gastric GIST, a laparoscopic subtotal gastrectomy was chosen instead of local excision. The surgery was performed under general anaesthesia, and five trocars were placed: a 10-mm umbilical camera port, a 10-mm port in the left and right upper quadrants and right iliac fossa, and a 5-mm port in the left iliac fossa. An exploratory laparoscopy was performed, with no evidence of regional lymphadenopathy and no visible ascites or hepatic and/or peritoneal metastases. Following the gastrectomy, a Billroth II reconstruction was performed. The surgery was uneventful.

The post-operative recovery was uneventful with close monitoring of the vital signs, fluid balance and possible complications (bleeding, infection or anastomotic dehiscence). The pain was managed with the use of analgesics (avoiding NSAIDs due to their potential to impair healing and increase bleeding risk) and thromboprophylaxis with low molecular weight heparin and early mobilization. In the immediate post-operative period, the patient remained on IV fluids and began clear liquids after 24-48 hours, gradually progressing to a soft, low-residue diet. The patient was discharged after seven days.

Endoscopic biopsy results took approximately nine days and showed no evidence of malignancy.

**Figure 3:**
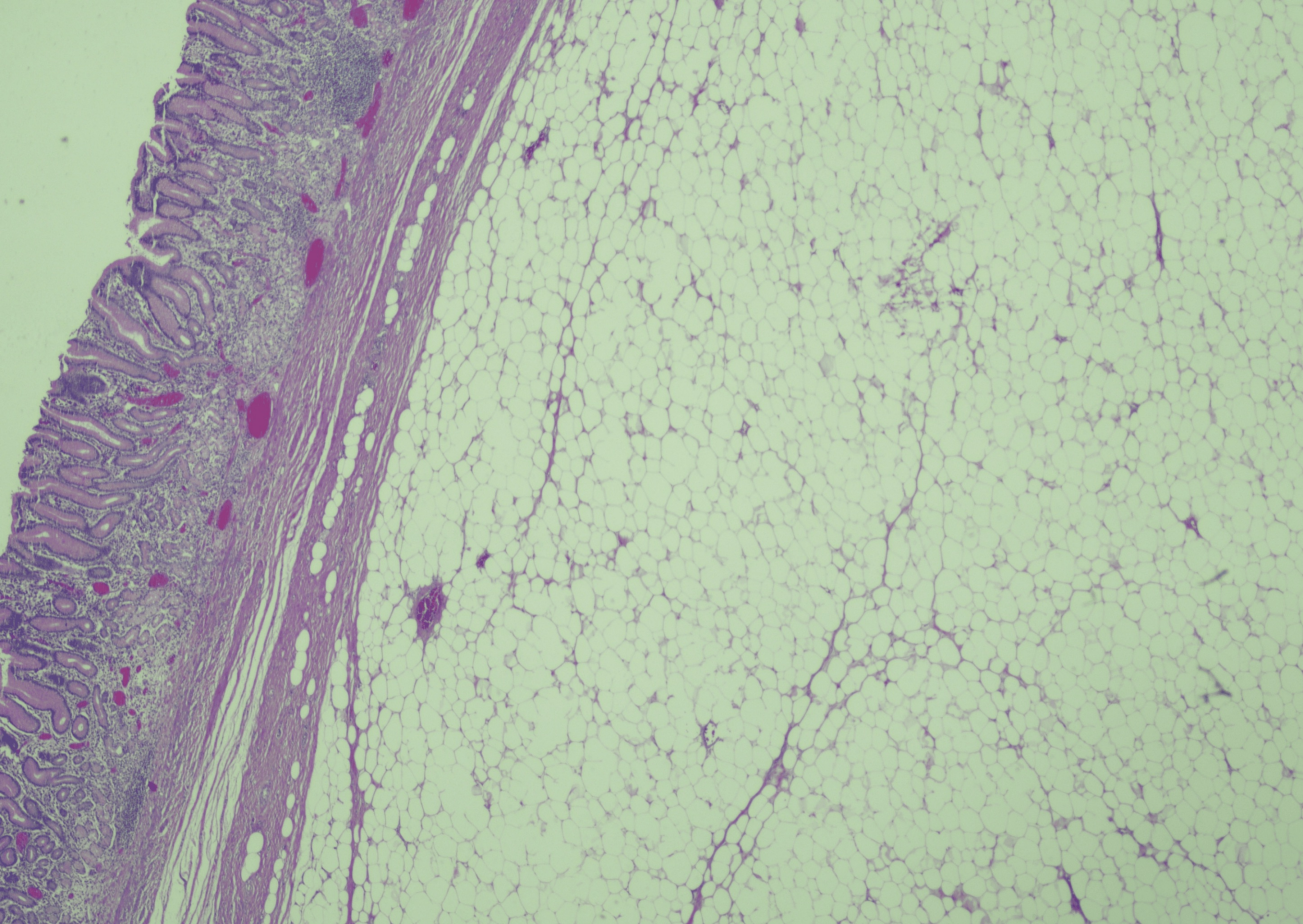
Histopathological examination, confirming a benign submucosal gastric lipoma, with absence of atypia or liposarcomatous features Haematoxylin and eosin (H&E) staining, 20× magnification.

Pathological examination revealed a yellow specimen measuring 85 × 45 × 37 mm with an ulcerated area, with histology confirming a benign submucosal gastric lipoma (Figure 3), with absence of atypia or liposarcomatous features. The overlying mucosa showed chronic inflammation and was negative for *Helicobacter Pylori*. The discrepancy between CT size (56 mm) and pathology size (85 mm) was interpreted as due to the partial volume effect or possibly specimen unfolding.

At one-month follow-up, she was tolerating a regular diet, and no complications were observed. A six-month laboratory tests and an upper gastrointestinal endoscopy showed no abnormal findings.

## Discussion

Gastric lipomas usually manifest between the fifth and seventh decades of life and are found mainly in women [1]. They are typically solitary (most often located in the antrum), slow-growing and asymptomatic lesions, but their size can lead to complications such as bleeding, obstruction, or perforation [3]. Some studies suggest that over half of individuals with lipomas over 2 cm in size may experience abdominal pain. In addition, approximately 37% of patients may exhibit symptoms such as chronic or acute gastrointestinal bleeding, obstruction, and dyspepsia [5]. In this case, the patient presented with significant anaemia and melena, attributable to ulceration overlying the large lipomatous lesion.

Imaging studies, particularly CT scans, are instrumental in characterizing submucosal gastric lesions. Lipomas generally display characteristic fat-density features, with attenuation values between -70 and -120 HU, aiding in differentiation from GISTs, which are usually soft tissue density tumours [6].

Endoscopic ultrasound (EUS) provides a more precise assessment of submucosal tumours, including details about their size and the specific layer of the gastric wall involved. This is especially useful for small tumours and those located in the subserosa, where conventional endoscopic biopsies are particularly challenging [1,7].

During upper gastrointestinal endoscopy, gastric lipomas appear as submucosal masses and are associated with characteristic signs that aid in diagnosis. The tenting sign occurs when the overlying mucosa can be easily lifted with biopsy forceps. The cushion sign is observed when gentle pressure with forceps creates a soft indentation [1,7].

In this patient, EUS was not performed due to its unavailability. Upper gastrointestinal endoscopy revealed a bleeding ulcerated mass, which was initially suspected to be a GIST or other submucosal neoplasm. Given the urgent context and the presence of low-flow haemorrhage, the decision was made to proceed with surgical intervention.

Management of gastric lipomas depends on size, location, symptomatology, and complication presence. Small, asymptomatic lipomas may be observed, while symptomatic or large lesions causing bleeding or obstruction typically require intervention [8]. Endoscopic resection is feasible for smaller lesions (<2 cm); however, larger tumours, especially those with ulceration or suspicion of malignancy, are often managed surgically.

Due to the absence of standardization in the surgical management and to the fact that preoperative diagnosis of gastric lipoma could not be established most of the time, most cases end with aggressive resections [1]. In this case, because of the size, active bleeding and diagnostic uncertainty, a laparoscopic subtotal gastrectomy was necessary, which resulted in complete removal and definitive diagnosis.

Histologically, they are composed of mature adipose tissue covered with a fibrous capsule. In this case, histopathology confirmed an ulcerated giant gastric lipoma, emphasizing that benign adipose tumours can reach considerable sizes and cause significant clinical manifestations, challenging the traditional perception of lipomas as small, incidental findings [6]. The differential diagnosis may include peptic ulcer disease, stromal tumour (GIST), liposarcoma [5], gastric adenocarcinoma and lymphoma.

Although gastric lipomas are benign tumours, there have been reports of simultaneous malignant lesions separate from the lipoma [9]. A proposed pathogenesis is that submucosal lipomas that extend into the lumen may cause repeated erosions or local inflammation of the gastric epithelium, predisposing to dysplasia and promoting gastric cancer [9]. Therefore, a complete preoperative evaluation is recommended to exclude an underlying malignant lesion.

## Conclusions

Giant gastric lipomas are rare benign tumours that can cause severe gastrointestinal bleeding due to ulceration, sometimes with hemodynamic repercussions. They can mimic other submucosal tumours on imaging and endoscopy, complicating diagnosis. Clinicians should consider gastric lipomas in the differential diagnosis of large submucosal gastric tumours presenting with bleeding. Surgical resection remains the mainstay of treatment for symptomatic lesions or when malignancy cannot be excluded. Awareness of this entity can facilitate appropriate management and optimize patient outcomes.
